# Functional Mental Disorders

**Published:** 1905-02-11

**Authors:** 


### HOSPITAL CLINICS.

### FUNCTIONAL MENTAL DISORDERS.

In the first of his Lettsonian lectures, Dr. Savage devoted himself to a consideration of the mental disorders for which no material pathology has yet been suggested. The subject touches all practitioners, for there are few men or women who do not at some period of their lives make personal acquaintance with one or more of the minor disorders of function. It is impossible here to reproduce the full interest of the address, which abounded in a quaint philosophy and in anecdotes which, impersonally considered, were as amusing as instructive.

Dealing with the common muscular and sensory disturbances of the hysterical state, Dr. Savage drew attention to the curious phenomenon of alternation; for instance, alternation of paraplegia with mania, of migraine and hay-fever with periods of definite insanity, of mania associated with the menstrual period with perfect sanity in the inter-menstrual epochs. He pointed out how many cases of what is called mental disorder represent merely a lack of harmony with the actual surroundings, as, for instance, when a man eminently fitted for the strenuous physical enterprise of an earlier civilisation is called upon to enter upon the humdrum spheres of a business existence, and refuses to keep himself as clean and tidy as his neighbours.

The neurotic he defined as those who evince too ready a response to their environment, and quoted the saying that "the world works on two wheels—the neurotic and the gouty." That many of the best-known names in history are those of neurotics there can be no doubt. No one can read Captain Mahan's "Life of Nelson" without being persuaded that that driving-wheel of the world, to continue the metaphor, was in the highest degree a neurotic one.

In conclusion, Dr. Savage indulged in an apt comparison well worth reproduction. If the human frame were like a clock, a mechanism of fixed elements adjusted in fixed surroundings for one fixed purpose, it would be fair to say that disturbances of function meant a material disorganisation of its parts. But man is a system needing, for the maintenance of what is called sanity, the widest possible range of adjustment to altered surroundings. Therefore it is unfair to dub insane a person who, instead of making the polite responses—to take an instance—which are demanded by conventional usage, replies with the first words that occur to him and scandalises his neighbours by his brutal incivility. He is not mad, but his range of accommodation is deficient. Mental accommodation is like that of the eye: its range is not the same in all individuals, and no ophthalmic surgeon considers a deficiency in accommodation in the light of an organic lesion.

